# Antiestrogen use reduces risk of cervical neoplasia in breast cancer patients: a population-based study

**DOI:** 10.18632/oncotarget.12957

**Published:** 2016-10-27

**Authors:** Chia-Jung Hsieh, Mun-Kun Hong, Pau-Chung Chen, Jen-Hung Wang, Tang-Yuan Chu

**Affiliations:** ^1^ Department of Public Health, Tzu Chi University, Hualien, Taiwan, ROC; ^2^ Department of Obstetrics and Gynecology, Buddhist Tzu Chi General Hospital, Hualien, Taiwan, ROC; ^3^ Institute of Medical Sciences, Tzu Chi University, Hualien, Taiwan, ROC; ^4^ Department of Public Health, National Taiwan University College of Public Health, Taipei, Taiwan, ROC; ^5^ Institute of Occupational Medicine and Industrial Hygiene, National Taiwan University College of Public Health, Taipei, Taiwan, ROC; ^6^ Department of Environmental and Occupational Medicine, National Taiwan University Hospital and National Taiwan University College of Medicine, Taipei, Taiwan, ROC; ^7^ Department of Medical Research, Buddhist Tzu Chi General Hospital, Hualien, Taiwan, ROC

**Keywords:** cervical neoplasia, antiestrogen, aromatase inhibitor, tamoxifen, cervical cancer

## Abstract

Estrogen has been proven to be a necessity for cervical carcinogenesis by transgenic mice studies. To determine whether long-term antiestrogens use could reduce the incidence of cervical neoplasia, a population-based cohort of 42,940 breast cancer patients with and without antiestrogen therapy were identified from the Taiwan National Health Insurance Database. All patients were followed for the most severe form of cervical neoplasia or until death. Their risks of cervical neoplasia were compared with Cox regression analysis and adjusted for age, Pap smear density and chemotherapy. Aromatase inhibitor (AI)-included antiestrogen users consistently exhibited a lower risk of low-grade cervical dysplasia [adjusted hazard ratio (HR) = 0.42, 95% CI 0.29 to 0.64, *P* < 0.0001] in the five-year follow-up analysis and in subgroup of regular Pap screenings (HR = 0.32, 95% CI, 0.20 to 0.50, *P* < 0.0001). A lower 10-year incidence of high-grade cervical dysplasia was also noted in the regular-screening group (HR = 0.49; 95% CI, 0.27 to 0.90; *P* = 0.0212), especially in the ≥ 50 years old group (HR = 0.34; 95% CI, 0.14 to 0.80; *P* = 0.014). The protection effect of Tamoxifen-only use for low-grade cervical dysplasia was only found in the young-age, regular-screening group (HR = 0.67; 95% CI, 0.48 to 0.93; *P* = 0.0167). In short, long-term use of AI-included antiestrogen conferred a lower risk of cervical neoplasia.

## INTRODUCTION

As the second most common malignancy in women worldwide, cervical cancer is the leading cause of cancer death in developing countries that lack organized Pap smear screening [1–3]. Epidemiologic studies have revealed that the long-term hormone exposure that occurs with high parity and oral contraceptive use is an independent risk factor for invasive cervical cancer (ICC) [4–5]. In unscreened populations of different countries, the incidence of ICC in women increases with age and plateaus in peri-menopause [6], suggesting a dependence on sex hormones. In the K14-HPV-E6/E7 transgenic mouse model of cervical cancer, an absolute requirement for estrogen and the estrogen receptor (ER) alpha has been proved in the full course of carcinogenesis [7, 8]. Cervical neoplasias of differing severities did not appear unless the mice were treated with a physiological level of 17β-estradiol and had a functional *Esr1* gene [9]. Importantly, the administration of a selective estrogen receptor modulator (SERM) led to the regression of the cervical neoplasias [10]. These findings suggest a pivotal role of estrogen and ERα in cervical carcinogenesis. However, evidence from human cervical carcinogenesis is lacking [11–13].

Aromatase inhibitor (AI) and tamoxifen are the two major antiestrogens that have been prescribed for more than two decades as adjuvant therapies [14, 15], and recently as chemoprevention agents for breast cancer [16]. We conducted a nation-wide, population-based study to determine whether antiestrogen use is associated with a lower risk of cervical neoplasia in breast cancer patients.

## RESULTS

**Figure 1:**
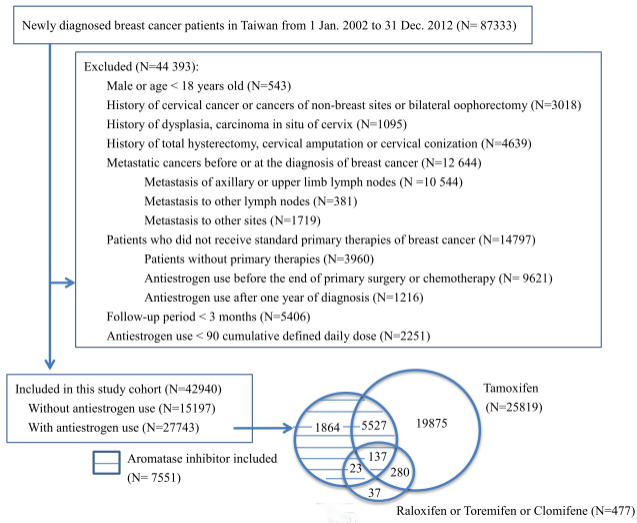
Selection of study population and status of antiestrogen use

We identified 87,333 eligible breast cancer patients who were registered for the first time in the RCIPD (Figure 1). After the exclusion criteria were applied, 42,940 patients were included in this study; 27,743 (64.6%) were antiestrogen users, and 15,197 (35.4%) were nonusers. Among the antiestrogen users, 25,819 (93.1%) had used tamoxifen, 7,551 (27.5%) had used aromatase inhibitor (AI-included users), 478 (1.7%) had used other SERMs, and 5,967 (21.5%) had used multiple antiestrogens during the study period (Figure 1). Among the AI-included users, 5,687 (75.3%) had ever used other SERMs and 1,864 (24.7%) used AIs only. The majority (91.1%) of the AI-included users used AI sequentially after discontinuing the use of a SERM. In the AI-included users, the median cDDD (interquartile range, IQR) use of AIs, SERMs, and tamoxifen were 468 (196 to 868), 252 (7 to 681), and 245 (1 to 672) respectively. AIs contributed to 67% (IQR, 31 to 99%) of the total cDDD of antiestrogens use in the AI-included users. The demographic characteristics of the groups are shown in Table 1. The AI-included users were generally older than the tamoxifen-only users and nonusers. The numbers of subjects identified across the years of the study were similar among the three groups. Greater proportions of nonusers (70.5%) and AI-included users (70.6%) had received chemotherapy compared to the tamoxifen-only users (54.1%). An average of 93.1% and roughly equal proportions of patients in each group had completed their primary therapy within 270 days after the diagnosis of breast cancer. The AI-included users had a slightly longer follow-up duration (median 4.11 years, IQR 2.29 to 5.00) than the tamoxifen-only users (median 3.75 years, IQR 1.82 to 5.00) and the nonusers (median 3.52 years, IQR 1.65 to 5.00). The mortality rate was higher in the nonusers and AI-included users (both 10.7%) than in the tamoxifen-only users (2.9%). A high proportion of antiestrogen users (81.4% in AI-included users and 74.1% in tamoxifen-only users) adhered to the medication for more than half of the cDDD in study period (days).

**Table 1:** Demographics and clinical characteristics of the breast cancer cohort with 5 year follow-up

Characteristic	All patients with breast cancer (*N*=42940)			Nonuser (*N*=15197, 35.7%)		AI-included user (*N*=7551, 17.7%)		Tamoxifen only user (*N*=19875, 46.6%)		*P*-value
	*N*	%		*N*	%	*N*	%	*N*	%	
SERM use	25879	60.3		—	—	5687	75.3	19875	100.0	—
Tamoxifen	25819	60.1		—	—	5664	75.0	19875	100.0	
Raloxifen	157	0.4		—	—	62	0.8	0	0.0	
Toremifen	250	0.6		—	—	96	1.3	0	0.0	
Clomifene	73	0.2		—	—	4	0.1	0	0.0	
AI-included use^a^	7551	17.6		—	—	7551	100.0	0	0.0	—
Age at breast cancer diagnosis (years)										< 0.0001
18-39	5549	12.9		2116	13.9	314	4.2	3045	15.3	
40-49	13929	32.4		4260	28.0	1750	23.2	7820	39.4	
50-59	12490	29.1		5088	33.5	2787	36.9	4562	23.0	
≥ 60	10972	25.6		3733	24.6	2700	35.8	4448	22.4	
Year of breast cancer diagnosis										< 0.0001
2002	2497	5.8		870	5.7	425	5.6	1162	5.9	
2003	2555	6.0		946	6.2	461	6.1	1107	5.6	
2004	2991	7.0		1068	7.0	600	8.0	1293	6.5	
2005	3662	8.5		1299	8.6	724	9.6	1605	8.1	
2006	3734	8.7		1283	8.4	781	10.3	1635	8.2	
2007	4176	9.7		1420	9.3	888	11.8	1839	9.3	
2008	4718	11.0		1616	10.6	928	12.3	2140	10.8	
2009	5126	11.9		1824	12.0	932	12.3	2340	11.8	
2010	5601	13.0		2012	13.2	877	11.6	2691	13.5	
2011	5747	13.4		2030	13.4	765	10.1	2935	14.8	
2012	2133	5.0		829	5.5	170	2.3	1128	5.7	
Chemotherapy for breast Cancer	26927	62.7		10718	70.5	5332	70.6	10742	54.1	< 0.0001
Breast cancer diagnosis to follow up start (day), median (IQR)	141 (21 to197)			143 (18 to 196)		160 (35 to 213)		130 (20 to 190)		< 0.0001
0-90	16153		37.6	5034	33.1	2324	30.8	8616	43.4	
91-180	13084		30.5	5360	35.3	2188	29.0	5467	27.5	
181-270	10749		25.0	3209	21.1	2495	33.0	4988	25.1	
271-360	2954		6.9	1594	10.5	544	7.2	804	4.1	
Follow up period (year), median (IQR)	3.76 (1.83 to 5.00)			3.52 (1.65 to 5.00)		4.11 (2.29 to 5.00)		3.75 (1.82 to 5.00)		< 0.0001
Mortality	3040		7.1	1627	10.7	806	10.7	583	2.9	< 0.0001
Antiestrogen adherence^b^										—
Nonuser	15197		35.4	15197	100.0	—	—	—	—	
<0.5	2038		4.8	—	—	1405	18.6	5145	25.9	
0.5-0.7	4625		10.8	—	—	1276	16.9	3227	16.2	
0.7-0.9	4555		10.6	—	—	1493	19.8	3459	17.4	
≥ 0.9	5012		11.7	—	—	3377	44.7	8044	40.5	
Ever Pap smear	29213		68.0	8895	58.5	5271	69.8	14807	74.5	< 0.0001
Pap smear every two years^c^	13389		31.2	3577	23.5	2174	28.8	7548	38.0	< 0.0001
Pap smear density^d^ (times/year), median (IQR)	0.40 (0.00 to 0.78)			0.22 (0 to 0.62)		0.40 (0 to 0.76)		0.55 (0 to 0.87)		< 0.0001
Antiestrogen accumulated dose (cumulative defined daily dose), median (IQR)										—
Antiestrogen	—			—		949 (532 to 1490)		—		
SERM						252 (7 to 681)		—		
Tamoxifen	—			—		245 (1 to 672)		742 (382 to 1311)		
AI^a^	—			—		468 (196 to 868)		—		
AI cDDD/Antiestrogen cDDD (%), median (IQR)	—			—		67 (31 to 99)		—		—

SERM: selective estrogen receptor modulator, AI: aromatase inhibitor; IQR: interquartile ranges, cDDD: cumulative defined daily dose

^a^AI: Exemestane or Letrozole or Anastrozole or Aminoglutethimide

^b^Antiestrogen adherence = cumulative defined daily dose/period of follow-up (day)

^c^Pap smear every two years: patients who had Pap smear at least once in every two years during follow-up

^d^Pap smear density = number of Pap smear/follow-up year

After adjusting for age, Pap smear density and chemotherapy (Table 3), the AI-included users exhibited a lower risk of low-grade cervical dysplasia than the nonusers [adjusted hazard ratio (HR) = 0.42, 95% confidential interval (CI), 0.29 to 0.64, *P* < 0.0001] in the five-year follow-up analysis. The hazard ratio was more prominent in the patients who had received Pap smears at least once every two years (HR = 0.32, 95% CI, 0.20 to 0.50, *P* < 0.0001), in the younger (18 to 49 years) group (HR = 0.23, 95% CI, 0.1 to 0.5, *P =* 0.0002), and also in the older (≥50 years) group (HR = 0.41, 95% CI, 0.23 to 0.72, *P* = 0.0019).

Similar results were found for high-grade dysplasia in AI-included users in the 10-year follow up (HR = 0.60, 95% CI, 0.39 to 0.93, *P* = 0.0231). A lower risk was also noted in the older (≥50 years) group (HR = 0.44, 95% CI, 0.24 to 0.79, *P* = 0.0058) in subgroup analysis. This phenomenon was also more prominent in the older (≥50 years) patient who received regular Pap smears at least once every two years in both the 5-year and 10-year follow-up analyses (HR = 0.30, 95% CI, 0.12 to 0.74, *P* = 0.0092 and HR = 0.34, 95% CI, 0.14 to 0.80, *P* = 0.014, respectively) (Table 3). Due to the limited event number, there was no association of antiestrogen use and the incidence of ICC in the 10-year follow-up (Table 2). The use of tamoxifen only revealed a marginal lower risk of cervical neoplasia. A lower risk of low-grade cervical dysplasia was noted in the young age (18 to 49 years) group that had undergone Pap smears at least once every two years (HR = 0.67, 95% CI, 0.48 to 0.93, *P* = 0.0167) (Table 3).

**Table 2:** Association between antiestrogens use and incidence of cervical neoplasia in the breast cancer cohort

	5 year follow-up						10 year follow-up					
	Low-grade dysplasia			High-grade dysplasia			High-grade dysplasia			ICC		
	Nonuser	AI-included	Tamoxifen only	Nonuser	AI-included	Tamoxifen only	Nonuser	AI-included	Tamoxifen only	Nonuser	AI-included	Tamoxifen only
No. of patients	15136	7516	19761	15076	7510	19660	14893	8084	19018	14843	8064	18935
Patients with event	113	33	200	53	27	99	63	30	103	13	10	20
Person-years	49154	26953	66633	49095	26942	66415	60919	38091	79764	60780	38025	79610
Incidence per 10^5^person-years	230	122	300	108	100	149	103	79	129	21	26	25
95% CI	191-276	86.8-172	261-344	82-141	68-146	122-181	80-132	55-112	106-156	12-36	14-48	16-38

HR: hazard ratio;

^a^Model adjusted for age, Pap smear density and chemotherapy

**Table 3:** Subgroup analysis of Cox's regression model for the association between antiestrogens use and cervical neoplasia

Modela		5 year follow-up								10 year follow-up			
	No. of Patients	Low-grade dysplasia				High-grade dysplasia				High-grade dysplasia			
		Event	HR	95% CI	*P*-value	Event	HR	95% CI	*P*- value	Event	HR	95% CI	*P*- value
All study population	42623	346				179				196			
Main model													
Nonuser	15197	113	1.0			53	1.0			63	1.0		
AI-included	7551	33	0.42	0.29-0.64	<0.0001	27	0.73	0.45-1.16	0.1780	30	0.60	0.39-0.93	0.0231
Tamoxifen only	19875	200	0.87	0.69-1.11	0.2661	99	0.94	0.67-1.33	0.7246	103	0.87	0.63-1.2	0.3943
Age, years													
18-49													
Nonuser	6376	62	1.0			22	1.0			25	1.0		
AI-included	2064	13	0.40	0.22-0.74	0.0031	13	1.19	0.59-2.38	0.6315	14	0.93	0.48-1.81	0.8298
Tamoxifen only	10865	119	0.75	0.55-1.03	0.0717	64	1.22	0.74-1.99	0.4374	67	1.15	0.72-1.83	0.5682
≥50													
Nonuser	8821	51	1.0			31	1.0			38	1.0		
AI-included	5487	20	0.45	0.27-0.76	0.0028	14	0.51	0.27-0.96	0.0359	16	0.44	0.24-0.79	0.0058
Tamoxifen only	9010	81	1.04	0.73-1.49	0.8319	35	0.74	0.45-1.21	0.2305	36	0.67	0.42-1.07	0.0922
Pap smear every two year	13299	296				128				135			
Main model													
Nonuser	3541	95	1.0			34	1.0			36	1.0		
AI-included	2157	24	0.32	0.20-0.50	<0.0001	12	0.43	0.22-0.83	0.0122	15	0.49	0.27-0.90	0.0212
Tamoxifen only	7455	177	0.78	0.61-1.01	0.0565	82	1.01	0.67-1.52	0.9556	84	1.01	0.68-1.50	0.9690
Age, years													
18-49													
Nonuser	1694	53	1.0			14	1.0			16	1.0		
AI-included	702	7	0.23	0.10-0.50	0.0002	6	0.73	0.28-1.93	0.5293	8	0.79	0.34-1.87	0.5952
Tamoxifen only	4680	108	0.67	0.48-0.93	0.0167	50	1.17	0.65-2.12	0.6042	51	1.07	0.61-1.89	0.8143
≥50													
Nonuser	1883	42	1.0			20	1.0			20	1.0		
AI-included	1472	17	0.41	0.23-0.72	0.0019	6	0.30	0.12-0.74	0.0092	7	0.34	0.14-0.80	0.0140
Tamoxifen only	2868	69	0.95	0.64-1.41	0.8034	32	0.90	0.51-1.59	0.7124	33	0.97	0.55-1.73	0.9253

**Figure 2:**
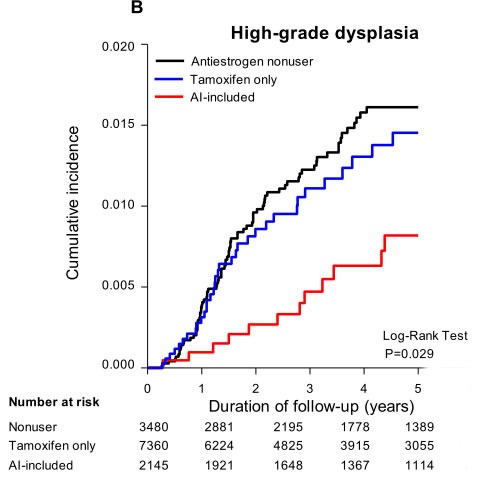
**Kaplan-Meier analysis for the association between antiestrogens use and A. low-grade dysplasia, B. high-grade dysplasia, for patients with Pap smear at least once every two year in 5 years follow-up analysis**.

The cumulative incidences of low-and high-grade cervical dysplasia are shown in the Kaplan-Meier analysis in Figure 2A and 2B. The AI-included users exhibited lower cumulative incidences of low- (log-rank test, *P* < 0.0001) and high-grade cervical dysplasia (log-rank test, *P* < 0.0029) compared to the nonuser and tamoxifen-only user groups.

## DISCUSSION

To our knowledge, this is the first population-based study to demonstrate that use of antiestrogen, especially the AI-included antiestrogen use, is associated with a lower incidence of cervical neoplasia. The protection against low grade cervical dysplasia was evident in a manner of no correlation to the age of subjects, whereas for high grade cervical dysplasia it was observed only in subjects ≥50 years old (Table 3, heterogeneity tests showed no different between subjects at 18-49 and ≥50 years old, *P* = 0.00541 and *P* = 0.0849 in 5- and 10-years analyses respectively in the main model; *P* = 0.0969 and *P* = 0.2358 in 5- and 10-years analyses in the regular pap screening subgroup, respectively, data not shown). This is in accordance with the natural histories of cervical tumorigenesis which displays an early onset of low grade dysplasia after HPV infection and a 2 to 5 years period to progress to high grade dysplasia which may last for more than one decade [17]. In accordance with the primary role of Pap screening in the detection of pre-invasive cervical lesions, the protective effect was even more obvious in the subjects who had received regular Pap smears. The sensitivity and specificity Pap smear for the detection of high-grade dysplasia or more severe cervical neoplasia in Taiwan is 81.9% and 98.6% respectively [18]. Meanwhile, the nation-wide 3-year screening rate increased from 14.5% in 1995 to 75.9% in 2014 [19]. In this population with breast cancer, an even higher screening rate was noted. A higher incidence of cervical neoplasias could be expected in these two user groups, but instead, we observed lower incidences. Thus, the hazard ratios for subgroup of “Pap smear at least once every two years” was more closely reflected the protective effects of antiestrogens on the occurrence of cervical neoplasias.

Tamoxifen use exhibited a marginal protection effect on cervical neoplasia, except in the subgroup of young patients with regular Pap smears, a lower risk of low-grade cervical dysplasia was noted (Table 3). This finding might be explained by the fact that tamoxifen acts as an ER agonist rather than antagonist in the uterus [20, 21]. The long-term use of tamoxifen in breast cancer patients actually increases the risks of endometrial and uterine stromal cancers [22]. However, studies of breast cancer patients who used tamoxifen did not reveal an increase in the incidence of abnormal cervical cytology [23]. Meanwhile, a phase 2 clinical trial reported a modest therapeutic effect of tamoxifen in recurrent non-squamous cell carcinomas of the cervix [13].

Although 78.7% of the AI-included users had ever used tamoxifen (Figure 1), the cDDD for tamoxifen was only 25.8% of the total cDDD antiestrogen in this group. Given that tamoxifen was neutral or only modestly effective in reducing the risk of cervical neoplasia, the protective effect of AI-included antiestrogen use can be attributed by the AIs. This result resembles the case of antiestrogen use in ER and/or progesterone receptor (PR)-positive breast cancer in which AI is more effective than tamoxifen in preventing recurrence of breast cancer [24]. Due to the limited number of cases with pure AI use (only 1,864 or 6.7% of all of the antiestrogens users), this study was not able to provide direct evidence regarding pure AI in the protection against cervical neoplasia.

This study has several strengths. First, this naturally occurring and passively followed cohort was devoid of selection or recall bias. Second, the use of antiestrogens in breast cancer patients was based on the expression of the ER and/or PR in the tumors, which is unrelated to the systemic health or the occurrence of cervical neoplasia. Third, because antiestrogens are essential for preventing the recurrence of breast cancer, drug adherence of the antiestrogen users was extraordinarily strong; 81.4% of AI-included users, and 74.1% of tamoxifen-only users exhibited adherences above half of the cDDD in the study period. Fourth, the main tool for outcome detections, the Pap smear, is sufficiently sensitive and specific and was widely applied in the study population. Finally, the 10-year follow-up period was sufficient to identify most of the occurrences of non-invasive cervical neoplasias. Due to the transient nature of low-grade cervical dysplasias and the fact that tamoxifen is typically used for five years after the primary therapy for breast cancer, we purposely set the analysis period to 5 years for low-grade dysplasia. In the analysis of high-grade cervical dysplasia, of which *in situ* carcinomas comprise a major portion, and the natural history is as long as 10 years or more, a longer follow-up is mandatory. Indeed, in the 10-year follow up, the AI-included users of postmenopausal ages exhibited a low risk (HR = 0.34, 95% CI, 0.14 to 0.80, *P* = 0.014) for high-grade cervical dysplasias. Additionally, the lack of ICC in the study population may due to relatively adequate screening that prevent the development of cancer.

There are also limitations of this study. As mentioned previously, the effect of AI was analyzed in the larger group of AI-included users and not in a group of pure AI users. Information regarding other confounders of cervical cancer, such as HPV infection status [2] [3], smoking [25], number of sexual partner [26] and sex hormone exposure (e.g. menopause, parity, and oral contraceptives pill use) was lacking. The smoking rate among Taiwanese women is as low as 4.3% [27]. Oral contraceptive use is also not popular in Taiwan. Therefore, the effect from these two factors may be minimal. Regarding to the major confounder of HPV infection, discrepant risks or prevalence of HPV infection may not present between groups, although the tamoxifen-only users, who exhibited a lower mortality rate, may have been more likely to maintain sexual activity and more prone to HPV infection.

In summary, this study demonstrated that antiestrogen use especially AI is associated with a reduction in cervical dysplasia.

## MATERIALS AND METHODS

### Study population and study design

The Taiwan National Health Insurance (NHI) database consists of health information from 23 million inhabitants since 1997. The database includes comprehensive disease diagnoses, hospital admissions, outpatient visits and prescription medications. Data for this study were obtained from the Registry for Catastrophic Illness Patient Database (RCIPD), which is a subset of the Taiwan NHI database that contains the complete medical records of all cancer patients. Due to the Registry of Catastrophic Illness, cancer patients receive medical care nearly free of charge while under NHI coverage. This database has been used extensively for epidemiologic research, and the information about diagnoses, prescription medications and hospitalizations is of reliable quality [28].

In this study, newly diagnosed breast cancer patients who were registered with the RCIPD from January 1, 2002 to December 31, 2012 were identified using the International Classification of Diseases, 9th Revision (ICD-9), codes 174.0 to174.9. Figure 1 shows the study design, exclusion criteria and the status of antiestrogen use in the study population. We excluded subjects who were male or younger than 18 years old, those with histories of cervical dysplasia, cervical carcinoma *in situ* (CIS), ICC and other cancers, those who had undergone hysterectomy, bilateral oophorectomy, cervical amputation, or cervical conization before or on the date of breast cancer diagnosis. We also excluded those with lymph node or distant metastases and subjects who did not receive standard primary therapy (an operation and/or chemotherapy) for breast cancer. To standardize the follow up protocol of the antiestrogen users and nonusers, those who had used antiestrogen before the end of standard primary therapy or one year after the diagnosis of breast cancer were excluded. The defined daily dose (DDD) advocated by the WHO was used to standardize the comparisons of drug usage between the different drugs, and the cumulative DDD (cDDD) of the antiestrogen users was calculated. Those with antiestrogen use that was less than 90 cDDD and whose follow-up periods were less than three months were also excluded.

The antiestrogens covered by the NHI during the study period for breast cancer adjuvant therapy included four SERMs (tamoxifen, raloxifen, toremifene and clomifene) and four AIs (anastrozole, letrozole, aminoglutethimide, and exemestane). To demonstrate the compliance with and consistency of the antiestrogen use, adherence was calculated by dividing the cDDD by duration of the follow-up period (days). To compare the effect of the different antiestrogens on cervical neoplasia, the patients were divided into three groups: nonusers, antiestrogen users who had ever used aromatase inhibitors, and antiestrogen users who had used tamoxifen only.

The follow-up scheme is shown in supplement (Figure S1). All breast cancer patients with or without antiestrogen use were followed after completion of the standard primary therapy for breast cancer. The follow up was initiated when the antiestrogen users began to use antiestrogen and when the nonusers had received the last primary therapy. All patients were followed until the occurrence of the most severe form of cervical neoplasias, the end of the study or death. Thus, if patient's medical records contained high-grade dysplasia and ICC during follow up period, only ICC was counted. The identification of ICC was based on a new registration of ICC in the RCIPD or by the diagnosis of ICC during hospitalization using the ICD-9 codes 180.0 to180.9. High-grade cervical dysplasia was identified by the diagnostic codes for cervical CIS (ICD-9 code 233.1) and cervical dysplasia (ICD-9 code 622.1) accompanied by the therapeutic procedure of cervical conization. Low-grade cervical dysplasia of the cervix was identified using the ICD-9 code 622.1 accompanied by procedures for cervical biopsy and/or endocervical curettage.

We explored several risk factors including age [6], Pap smear density [29], and chemotherapy treatment [30] that might have interfered with the association between antiestrogen use and the risk of cervical neoplasia. Pap smear density was calculated as the number of Pap smears divided by the number of follow-up person-years. This study was approved by the Research Ethics Committee of the medical center Hualien Tzu Chi Hospital, Hualien, Taiwan (IRB101-98).

### Statistical analysis

Based on a Cox proportional hazards model, the hazard ratios (HRs) and accompanying 95% confidence intervals (CIs) were computed with adjusting for age at the time of breast cancer diagnosis, Pap smear density and chemotherapy. Since death is a competing risk for loss to follow-up, we also did a competing risk analysis by Fine and Gray method [31]. The consistencies and differences in the risk of cervical neoplasia were evaluated by conducting subgroup analyses based on age and Pap smear. To test whether excluding subjects whose follow-up periods were less than three months would cause selection bias, sensitivity analyses of including all the subjects or excluding the subjects with different follow-up period were conducted (Table S1). The cumulative incidence of cervical neoplasia was plotted using the Kaplan-Meier method, and the differences between the curves were tested with the log-rank test. The occurrence of cervical dysplasia is always identified by Pap screening [18], therefore, the cumulative incidence of cervical dysplasia was analyzed in the subgroup of patients who underwent Pap smears at least once every two years. The statistical analyses were performed using SAS, version 9.4 (SAS Institute, Inc., Cary, NC) and all tests were two-sided.

## SUPPLEMENTARY MATERIALS FIGURES AND TABLE



## Abbreviations

AI: aromatase inhibitor
HR: hazard ratio
CI: confidence interval
HPV: human papilloma virus
ICC: invasive cervical cancer
ER: estrogen receptor
SERM: selective estrogen receptor modulator
NHI: National Health Insurance
RCIPD: Registry for Catastrophic Illness Patient Database
CIS: cervical carcinoma *in situ*
DDD: defined daily dose
