# Supplementary material for: Antiestrogen use reduces risk of cervical neoplasia in breast cancer patients: a population-based study
Source: Oncotarget. 2016 Oct 27;8(17):29361–9. doi: 10.18632/oncotarget.12957 (PMC5438736; doi:10.18632/oncotarget.12957)
Supplement: Supplementary file 1 [file oncotarget-08-29361-s001.pdf]

Antiestrogen use reduces risk of cervical neoplasia in breast cancer patients: a population-based study

Supplementary Material

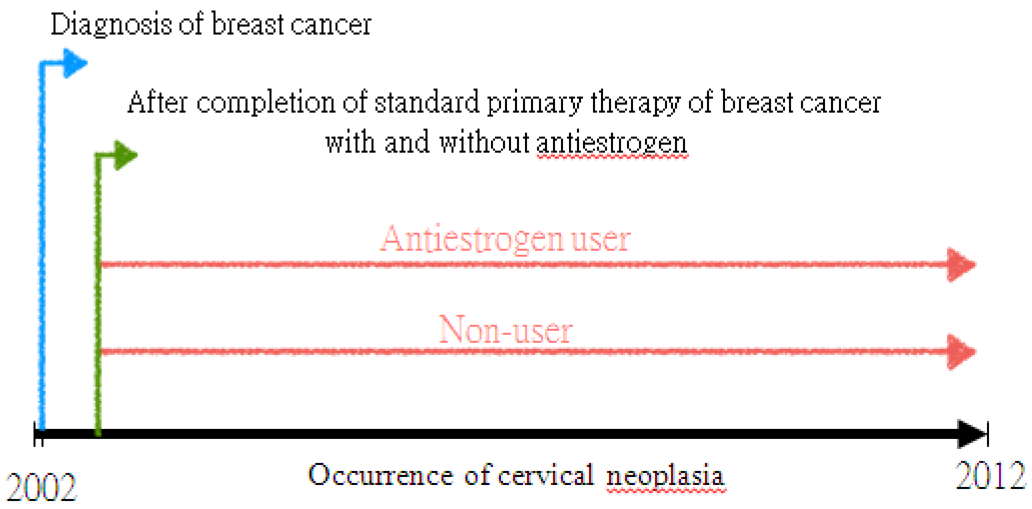

Figure S1. Schema of study design and follow up

Table S1. Sensitivity analysis of Cox's regression model for the association between antiestrogens use and cervical neoplasia

Model[a]	5 year follow-up								
	No. of patients	Low-grade dysplasia				High-grade dysplasia			
		Event	HR	95% CI	P-value	Event	HR	95% CI	*P*-value
**Pap smear every two year**									
**Main model** (Exclude those follow-up period were less than 3 months)	13299	296				128			
Nonuser	3579	95	1.0			35	1.0		
AI-included	2179	24	0.33	0.21-0.51	<0.0001	12	0.41	0.21-0.81	0.0093
Tamoxifen only	7558	177	0.80	0.62-1.04	0.0980	82	0.99	0.66-1.47	0.9408
Exclude those follow-up period were less than 2 months	13411	309				132			
Nonuser	3649	101	1.0			35	1.0		
AI-included	7584	25	0.34	0.22-0.53	<0.0001	12	0.42	0.22-0.81	0.0101
Tamoxifen only	2178	183	0.87	0.68-1.12	0.2907	85	1.02	0.68-1.52	0.9309
Exclude those follow-up period were less than 1 month	13483	319				134			
Nonuser	3713	108	1.0			36	1.0		
AI-included	7592	186	0.33	0.21-0.51	<.0001	86	0.43	0.22-0.84	0.0132
Tamoxifen only	2178	25	0.87	0.68-1.11	0.2513	12	1.06	0.71-1.58	0.7836

Include those follow-up period were less than 3 months	13654	325				136			
Nonuser	3884	114	1.0			38	1.0		
AI-included	7592	186	0.32	0.21-0.5	<0.0001	12	0.43	0.22-0.84	0.0129
Tamoxifen only	2178	25	0.83	0.65-1.06	0.138	86	1.06	0.71-1.58	0.7782

HR: hazard ratio, AI: aromatase inhibitor

[a]Model adjusted for age, Pap smear density and chemotherapy

Table S2. Subgroup analysis of Cox's regression model for the association between antiestrogens use and total cervical neoplasia

Model[a]		5 year follow-up				10 year follow-up			
	No. of Patients	Total Cervical Neoplasia				Total Cervical Neoplasia			
		Event	HR	95% CI	*P*- value	Event	HR	95% CI	*P*- value
**All study population**	42623	556				658			
**Main model**									
Nonuser	15197	174	1.0			214	1.0		
AI-included	7551	68	0.56	0.42-0.75	<0.0001	85	0.51	0.39-0.65	<0.0001
Tamoxifen only	19875	314	0.90	0.75-1.10	0.3056	359	0.88	0.74-1.05	0.1488
Age, years									
18-49									
Nonuser	6376	86	1.0			110	1.0		
AI-included	2064	28	0.64	0.41-0.98	0.0388	36	0.53	0.36-0.78	0.0012
Tamoxifen only	10865	187	0.87	0.67-1.13	0.2909	216	0.83	0.66-1.05	0.1286
≥50									
Nonuser	8821	88	1.0			104	1.0		
AI-included	5487	40	0.52	0.36-0.76	0.0007	49	0.49	0.35-0.69	<0.0001
Tamoxifen only	9010	127	0.95	0.72-1.25	0.7014	143	0.94	0.73-1.22	0.6514
**Pap smear every two year**	13299	442				503			
**Main model**									

Nonuser	3541	131	1.0			150	1.0		
AI-included	2157	41	0.39	0.28-0.56	<0.0001	57	0.46	0.33-0.62	<0.0001
Tamoxifen only	7455	270	0.87	0.70-1.07	0.1933	296	0.87	0.71-1.06	0.1619
Age, years									
18-49									
Nonuser	1694	67	1.0			81	1.0		
AI-included	702	14	0.36	0.20-0.64	0.0005	26	0.50	0.32-0.79	0.0025
Tamoxifen only	4680	160	0.78	0.58-1.04	0.0882	176	0.75	0.57-0.98	0.0324
≥50									
Nonuser	1883	64	1.0			69	1.0		
AI-included	1472	27	0.43	0.27-0.67	0.0002	31	0.44	0.29-0.68	0.0002
Tamoxifen only	2868	110	0.99	0.72-1.35	0.9283	120	1.05	0.78-1.43	0.7417

HR: hazard ratio;

[a]Model adjusted for age, Pap smear density and chemotherapy

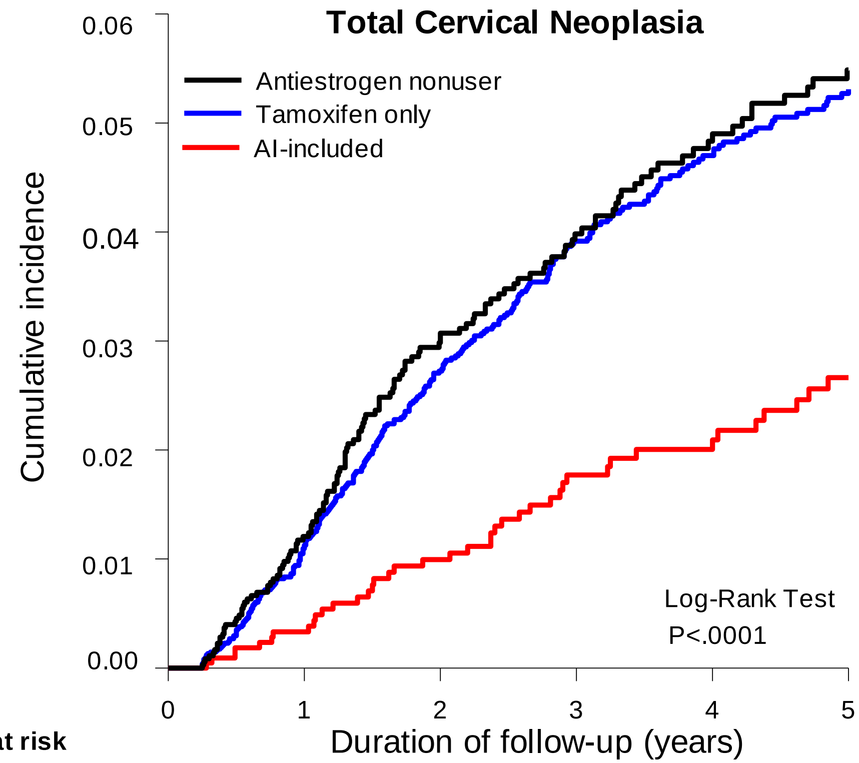

Number at risk

Nonuser	3577	2948	2229	1796	1396	1169
Tamoxifen only	7548	6360	4901	3948	3072	2485
AI-included	2174	1945	1663	1373	1119	907

Figure S2. Kaplan-Meier analysis for the association between antiestrogens use and total cervical neoplasia for patients with Pap smear at least once every two year in 5 years follow-up analysis.
